# HERPETIFORM PEMPHIGUS CLINICALLY RESEMBLING BULLOUS PEMPHIGOID

**DOI:** 10.4103/0019-5154.43218

**Published:** 2008

**Authors:** Vandana Mehta, C Balachandran, Sudhir Nayak

**Affiliations:** *From the Department of Skin and STD, Kasturba Medical College, Manipal, Karnataka, India. E-mail: vandanamht@yahoo.com*

Herpetiform pemphigus was first introduced by Jablonska in 1975, as a variant of pemphigus that combines the clinical features of dermatitis herpetiformis with the immunological features of pemphigus.^1^ Because of its rare and atypical clinical presentation, patients are often initially diagnosed as Dermatitis herpetiformis, Linear IgA bullous disease, Bullous pemphigoid or Pemphigus foliaceus. We report a case of herpetiform pemphigus in an elderly lady which was initially misdiagnosed as bullous pemphigoid, however histology and immunofluorescence helped in clinching the diagnosis.

**Fig. 1:**
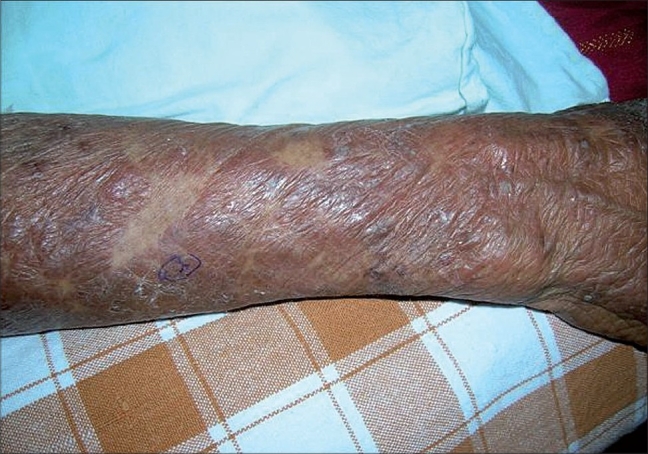
Grouped flaccid vesicles and bullae in a herpetiform distribution

A 75-year-old woman presented with a one year history of an intensely pruritic eruption which began on her extremities and later expanded to involve the trunk with only partial response to topical corticosteroids and antihistamines. She was otherwise healthy with no accompanying constitutional symptoms and not on any other medications. Cutaneous examination revealed a symmetric polymorphous eruption of old and new lesions, comprising of erythematous urticarial papules and plaques studded with clear and cloudy vesicles (Fig. 1), ulcerated and crusted lesions arranged in herpetiform groups. Oral and genital mucosa were normal. Clinically a differential diagnosis of bullous pemphogoid was entertained, however a Tzanck smear from a clear vesicle showed abundant neutrophils, with only few eosinophils. Biopsy for histopathology showed mild epidermal hyperplasia with a subcorneal blister containing neutrophils and few acantholytic cells. Predominant areas of neutrophilic spongiosis with a superficial perivascular infiltrate of eosinophils and neutrophils were seen in the papillary dermis. Direct and indirect immunofluorescence demonstrated strong intercellular deposits of IgG and C3 in the upper epidermis. Based on the histology and immunofluorescence findings, the diagnosis was revised to herpetiform pemphigus and patient was started on oral steroids.

Pemphigus herpetiformis (PH) is recognized as a distinct subset of pemphigus by its pruritus, rarity of mucosal involvement, invariable presence of eosinophils on histology and its tendency to respond to sulfones. Various terms that have been used to describe it are *acantholytic herpetiform dermatitis*, *pemphigus controlled by sulfapyridine*, *mixed bullous disease* and *eosinophilic spongiosis in pemphigus*. Clinically patients present with severe intractable pruritus and erythematous urticarial papules and plaques with occasional vesicles in a herpetiform arrangement. Even though the clinical picture is reminiscent of dermatitis herpetiformis, the histological findings are variable and include eosinophilic spongiosis, subcorneal pustules with minimal or no apparent acantholyis.^2^ Though eosinophilic spongoisis is classically seen in pemphigus herpetiformis, neutrophilic spongiosis may also be encountered and should be recognized as an important diagnostic clue^3^ as was seen in our case. Definitive diagnosis is established by immunopathology which shows *in vivo* bound IgG deposits on the keratinocyte cell surface primarily in the upper epidermis. The auto-antibodies in most patients of PH target desmoglein 1 and because desmoglein 1 is predominantly located in the upper epidermis, there is preferential binding of IgG to the upper epidermis as was seen in our case. Why the auto-antibodies in PH and pemphigus vulgaris give rise to different clinical presentations could be explained by their preferential binding to different epitopes on the same antigen molecule.^4^

In our case, the presence of severe pruritus in an elderly female with blisters and absence of mucosal involvement made us think of bullous pemphigoid, however, to our surprise the histology and immunofluorescence turned out to be PH. Patient was started on oral steroids along with dapsone and is currently under follow-up.
